# Non-surgical Management of Periapical Lesions With the Use of Newer Modalities in Adjunct to the Conventional: A Case Series

**DOI:** 10.7759/cureus.57314

**Published:** 2024-03-31

**Authors:** Simran Kriplani, Shweta Sedani, Abeer Mishra, Utkarsh Umre

**Affiliations:** 1 Department of Conservative Dentistry and Endodontics, Sharad Pawar Dental College and Hospital, Datta Meghe Institute of Higher Education and Research, Wardha, IND

**Keywords:** ozonated olive oil, periapical lesion, triple antibiotic paste, ciprofloxacin and minocycline, metronidazole

## Abstract

For endodontic therapy to be successful, the root canal space must be sterilized. This is often done using intracanal irrigants and medications. To accomplish periradicular region sterilization and healing, various intracanal medicaments and irrigation techniques have been researched for better treatment outcomes. Ozonated olive oil is the most researched and successful adjunct to other medicaments owing to its antibacterial properties. Triple antibiotic paste (TAP) (metronidazole, ciprofloxacin, and minocycline) was incorporated as an inter-appointment intracanal dressing. Currently, many newer advances are depicting synergistic effects in the elimination of persistent endodontic pathogens. Given this, in the current case series, periapical lesions were managed non-surgically for alternating weeks by the advent of triple antibiotic paste (TAP) and ozonated olive oil (O_3_-oil) with laser activation. Irrigation and its effects were further enhanced with the use of a laser, aiming for thorough debridement and rendering the canal free of microbes. Once the patient was asymptomatic and there was no sinus drainage seen, final obturation was done. Therefore, this case series depicts that traditional root canal therapy with the use of ozonated olive oil and laser activation can non-surgically heal the lesion, leading to successful treatment outcomes. Periapical lesions in three cases have been observed; on the initial visit, all lesions were accessible, cleansed, and shaped. We administered ozonated olive oil with laser activation and a triple antibiotic paste on the following visit. In all three cases, six-month follow-ups have shown evidence of a successful course of therapy.

## Introduction

It has proven difficult for the clinician to eradicate the infection when there are extensive periapical lesions. Because of the polymicrobial nature of the root canal system, sterilization is difficult. Because the intraradicular load in the canal has not been sufficiently cleaned out, it fails root canal therapy and causes recurring infections [1]. In vitro and in vivo research highlights adding antimicrobial medications in conjunction with mechanical cleaning, which improves treatment outcomes to sterilize and mend diseased root dentin [2]. In most cases, periapical lesions are an inflammatory reaction to bacteria and their byproducts invading the root canal system. Therefore, these lesions can subside through apoptotic pathways if the lesion is adequately cleared of inflammatory exudates to lower the hydrostatic pressure and if non-surgical root canal therapy eliminates the microbiological etiology.

The Niigata University Cardiology Research Unit conducted studies on lesion sterilization and tissue repair (LSTR) treatment. It comprises the management of infectious lesions, including periradicular, pulpal, and dentinal diseases, using a mixture of antibacterial medications (ciprofloxacin, minocycline, and metronidazole) [3]. Ozone has been utilized in medicine over the past ten years because of its strong biocompatibility, antiviral, antifungal, and antibacterial properties. Ozonated oils have been used topically to treat mucosal infections and chronic ulcers. Ozonated olive oil has been utilized to treat post-extraction alveolitis because of its strong germicidal and oxygenating properties against various bacteria, which promote tissue repair and regeneration [4].

## Case presentation

Case 1

**Figure 1:**
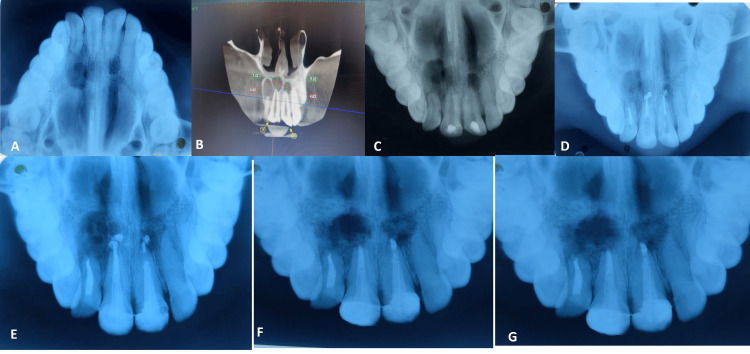
(A) Occlusal radiograph; (B) cone beam computed tomography of tooth number 11, 12, 21; (C) access opening done with tooth number 11, 12, 21; (D) apical plug was placed with tooth number 11, 12, 21; (E) fiber post placement was done with tooth number 11, 12, 21; (F) six-month follow-up radiograph; and (G) a 12-month follow-up radiograph.

A female aged 25 years old visited the Department of Conservative Dentistry and Endodontics with a complaint of pain and pus discharge in the upper right back region of the jaw. She had an insignificant medical history and was ruled out with a detailed drug history or any hereditary disorder. The patient gives no history of any previous dental treatment. Ellis Class III fracture was seen with 11, 12, and 21; while they showed no mobility, they were slightly tender to percussion when probed. There was a negative response from the electric pulp test. Pulp exposure of the involved teeth was evident on an occlusal radiograph. For further confirmation, cone beam computed tomography was advised (Figure 1).

During the same visit, we placed the rubber dam, created an access opening, and removed the hemorrhagic, purulent exudates from the canal to initiate the root canal therapy procedure. We used an apex locator to ascertain the length of the root canal and validated it with a radiograph. Using a step-back method, biomechanical preparation was carried out with rotary NiTi files until F1 (6% taper). After each instrumentation session, a 27-gauge endodontic needle was used to aggressively irrigate the canal with a 3% sodium hypochlorite solution. Then, after applying a dressing of calcium hydroxide and drying the canals with sterile paper points, temporary closure was carried out. Three times a week, the calcium hydroxide dressing was replaced. The canals' discharge did not entirely stop after three weeks.

The course of therapy was altered. The same procedure was used to irrigate root canals, which were thoroughly dried. Next, a TAP (ciprofloxacin, metronidazole, and minocycline, 100 mg of each drug in 0.5 ml of the total solution) was put in with the aid of a lentulo spiral. Then the ozonated olive oil was used for better efficacy, and laser activation was done, called ozonated laser activation therapy (OLAT). For a month, dressings were changed once a week to feel no discomfort when percussion was done. After adopting a lateral compaction technique to obturate with gutta-percha, the final irrigation was carried out using 2% chlorhexidine. Post-endodontic treatment composite restoration was done. The patient was prescribed adjunctive antibiotic therapy pre-operatively as Augmentin-625 mg, Zerodol-SP, and Pantoprazole 40 mg twice daily for five days. In the post-treatment course, the patient was prescribed Zerodol-SP for symptomatic relief and advanced healing.

Case 2

A 19-year-old girl complained of discomfort in the area of her upper left back teeth and compliance with sinus discharge in her teeth numbers 14 and 15. She visited the Department of Conservative Dentistry and Endodontics. The patient gave no significant medical or dental history; she has not undergone any dental procedures in the past. Following radiological assessment, a cone beam computed tomography assessment showed periapical radiolucency involving both roots, each measuring 3 x 3 mm. Heat and electric pulp tests failed to produce any response in the tooth. It was diagnosed as pulp necrosis with a periapical abscess in 14; 15 showed a delayed response suggestive of asymptomatic irreversible pulpitis. Incision and draining of the abscess, then non-surgical endodontic therapy using ozonated olive oil for laser activation and triple antibiotic paste (TAP) as an intracanal medication.

**Figure 2:**
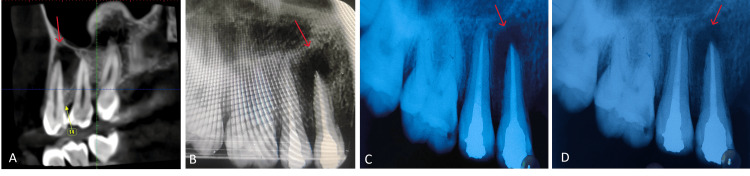
(A) Cone beam computed tomography of tooth number 14, 15; (B) post-obturation and final restoration with tooth number 14, 15; (C) six-month follow-up radiograph; and (D) 12-month follow-up radiograph.

A No. 11 BP blade was used to make the incision on the initial visit, and the pus was then drained. The access opening under rubber dam isolation was followed by a thorough biomechanical preparation using rotating ProTaper files. Ten milliliters of 2.5% sodium hypochlorite per canal were used for irrigation, which was followed by saline irrigation and an open dressing. Triple antibiotic paste (TAP) was inserted intracanally on the subsequent appointment. After receiving ozonated laser activation treatment and occasional dressing changes, the patient was instructed to return after one week. Consequently, a single-cone obturation method was used to seal the canals, once the root canal process was finished. With Amalgam, the final restoration was completed. Three and six months later, the patient was contacted for follow-up radiographs, which are shown in Figure 2.

Case 3

**Figure 3:**
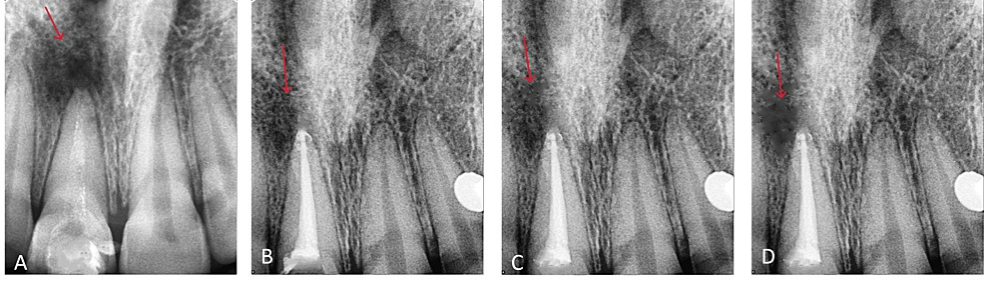
(A) Pre-operative intra-oral periapical radiograph with tooth number 11; (B) post-obturation radiograph with tooth number 11; (C) six-month follow-up radiograph; (D) 12-month follow-up radiograph.

A 22-year-old woman complained five years ago of having a cracked tooth in the upper front area of her jaw. The medical and dental history was insignificant and had no history of previous dental treatment. Clinical examination indicated intact tooth structures with healthy gingiva and normal probing depths in tooth number 11. Intra-oral periapical (IOPA) revealed intact dentin and enamel in conjunction with periapical radiolucency. The tooth showed a delayed reaction to pulp sensitivity testing. Tooth number 11 was determined to be asymptomatic, irreversible pulpitis with apical periodontitis. Ozonated laser activation therapy was planned as a non-surgical endodontic treatment. Cleaning and shaping of the canals using rotary ProTaper files were carried out after access opening under rubber dam isolation and working length calculation. 10 mL of 2.5% sodium hypochlorite per canal was used for irrigation, followed by saline irrigation and a final rinse with 17% ethylenediamine tetraacetic acid (EDTA). During the same appointment, triple antibiotic paste was inserted intracanally since the canals were not producing any discharge. After treatment completion, the patient was requested to report and was given a temporary dressing, with a follow-up call scheduled periodically. The triple antibiotic paste was completely removed before the obturation process using the lateral condensation approach was finished. The composite was used for the anterior buildup and definitive restoration. As seen in Figure 3, in periapical radiography, to determine if the patient had any signs or symptoms, they were assessed clinically and radiographically. 

## Discussion

The initial treatment in this trial was calcium hydroxide; however, it did not affect the symptoms. A triple antibiotic paste was substituted for the original therapy regimen. Following its use, the symptoms disappeared. Antibacterial drugs aid in the sterilization of the diseased root dentin, especially the deep layers, where the bulk of the bacteria causing the infection are obligatory anaerobes in the root canal wall's deep layers. These substances penetrate deeper, diseased dentinal layers. Several case studies demonstrate how well non-surgical therapy works for having an ongoing sinus tract and a non-vital pulp. Triple antibiotic paste helps the periapical tissue repair and regenerate [5].

After using antibiotic paste and irrigation, Windley et al. found a significant decrease in bacterial colonies. After 10 milliliters of 1.25% sodium hypochlorite irrigation, almost 90% of the bacteria were still positive. However, after two weeks of triple antibiotic paste treatment (TAP), this decreased to 30% [6]. In non-vital young permanent teeth, Bose et al. compared the intracanal medications formocresol, calcium hydroxide, and TAP. Compared to either of the groups, the dentin thickness of the wall increased by a significant percentage in the triple antibiotic group. TAP can support the pulp-dentin complex's functional development [7].

Among antibacterial medications, metronidazole was decided upon as the preferred option. Since the periradicular lesion's bacterial ecology is complicated, metronidazole may not be enough to eradicate all of the germs, meaning other medications are possibly required to disinfect the infected root canal. Therefore, additionally, metronidazole, ciprofloxacin, and minocycline were required to clean the contaminated root canal system [8]. Large periapical lesions were treated with TAP by several researchers, who reported their outcomes in the articles. Apical lesions were treated with a conservative approach without surgical intervention [9]. TAP has demonstrated efficacy when used in patients with an intracanal disinfectant in the treatment of aggressive lesions present periodically and also in regenerative endodontic procedures. Iwaya et al. decontaminated immature teeth with necrosed pulp using a combination of irrigation solution and TAP, and they saw the dentin wall thicken and the apical aperture and periapical lesion recover [10].

Periapical lesions are now treated either surgically or non-surgically. In the therapy of periapical lesions, surgery was the rule of thumb when the lesion size was very large. The ability of the pulp-periapical lesion to heal on its own has, however, favored a non-surgical treatment due to advances in scientific understanding of the origin, pathologic character, and clinical behavior of endodontic periapical lesions. It has been shown that when treated conservatively with non-surgical endodontic treatment, up to 94.4% of periapical lesions exhibit partial or full recovery [11,12]. Ozone has a high degree of biocompatibility and is a powerful antioxidant with few side effects. Research has demonstrated that it may effectively interact with and eradicate the microorganisms in the system of root canals. As an antimicrobial agent, ozone has 1.5 times the effectiveness of chlorine against a variety of microbes, indicating its strong oxidation potential [13]. Ozonated oils are the result of the chemical reaction between ozone and the unsaturated fatty acids present in vegetable oils [14]. Only the carbon-carbon double bonds present in unsaturated fatty acids allow ozone to react with oil, resulting in a variety of hazardous byproducts, including aldehydes, hyperoxides, ozonides, peroxides, and polyperoxides, among other oxygenated molecules [15]. The broad range of antibacterial action of ozonized oil is caused by these molecules [16]. Root canal treatments can be used to treat large cyst-like periapical lesions with success. Osteoarthritis may be adversely affected by pre-operative cortical bone deficiency and the apical degree of obturation [17,18].

## Conclusions

This case study shows that periapical lesions can be effectively controlled without surgery, and recovery can be speeded up by using intracanal medications (triple antibiotic paste) in conjunction with traditional root canal therapy and ozonated laser-activated therapy. The antibacterial activity of ozonated olive oil plays a critical role in this process. Large periapical lesions can be successfully managed using a non-surgical root canal treatment that uses three-dimensional root canal obturation and intracanal medication. As such, it has to be the initial therapeutic option that is chosen.
